# Graphene-Like Layers from Carbon Black: In Vivo Toxicity Assessment

**DOI:** 10.3390/nano10081472

**Published:** 2020-07-27

**Authors:** Marta d’Amora, Michela Alfe, Valentina Gargiulo, Silvia Giordani

**Affiliations:** 1 Nano Carbon Materials, Istituto Italiano di Tecnologia (IIT), 16163 Genoa, Italy; marta.damora@iit.it; 2 Institute for Research on Combustion (IRC)-CNR, 80126 Naples, Italy; alfe@irc.cnr.it (M.A.); v.gargiulo@irc.cnr.it (V.G.); 3 School of Chemical Sciences, Dublin City University (DCU), Glasnevin, D09 C7F8 Dublin, Ireland

**Keywords:** graphene-like layers, graphene related materials, toxicity, zebrafish

## Abstract

Graphene-like (GL) layers, a new graphene-related material (GRM), possess peculiar chemical, colloidal, optical and transport properties. Considering the very recent promising application of GL layers in biomedical and bioelectronic fields, it is of utmost importance to investigate the toxicological profile of these nanomaterials. This study represents an important first report of a complete in vivo toxicity assessment of GL layers on embryonic zebrafish (*Danio rerio*). Our results show that GL layers do not lead to any perturbations in the different biological parameters evaluated, indicating their good biocompatibility on a vertebrate model. The new insight into the biosafety of GL layers will expand their applications in nanomedicine.

## 1. Introduction

Graphene-related materials (GRM) are widely used nanomaterials in the electronic [1,2], energy [3,4,5] and environmental fields [5,6], thanks to their exceptional optical, electrical and thermal properties [7,8,9,10,11]. Recently, the high surface area and the possibility of chemical and physical modifications of GRM have promoted their employment into biological and biomedical applications, ranging from microbial disinfection to medical devices. Among the required characteristics of biomedical nano-platforms, good biocompatibility is a key characteristic. In this framework, different reports have explored the potential harmful bio-effects of several graphene nanostructures on in vitro and in vivo models, revealing the interactions of different graphene-based nanomaterials at cellular and tissues interfaces [12,13,14,15,16,17,18,19]. The exposure of GRM to different cellular models induced toxicity in most cases, due to the interactions of GRM with several biomolecules, such as DNA, proteins and components of the cellular membrane [20]. In particular, these interactions damage the biomolecular structures, causing their degradation or denaturation [21,22]. These harmful effects were reported prevalently in endothelial cells, red blood cells and macrophages [22]. To understand the possible chronic effects of GRM, different in vivo toxicological screenings were carried out in zebrafish (*Danio rerio*) and mouse models. The interactions between different graphene-related nanomaterials and zebrafish caused several perturbations in the toxicological endpoints, in particular an increase in the mortality rate and delay in the chorion aperture (hatching rate) [17,19,23,24,25,26,27,28,29,30,31,32,33]. Among the graphene family, mainly graphene oxide (GO) and nanographene oxide (NGO) were investigated in terms of toxicity in zebrafish. GO and NGO exposed to embryos by different approaches caused a high incidence of malformations affecting the tail, the eye, the heart and the yolk sac [34,35,36,37,38,39]. Recently, we also demonstrated that a commercial GO at high concentrations caused harmful effects on the embryogenesis [40], while laser-induced graphene (LIG) presented good biocompatibility in the same developmental stages [41]. Finally, various degrees of toxicity in rats and mice have been reported for nanomaterials of the graphene-family, including effects on the nervous systems and the lungs [18,42,43,44].

Recently, a new class of materials belonging to the GRM family, the graphene-like (GL) layers [45], has shown peculiar chemical-physical properties, making them promising nanomaterials (as they are [46] or combined to produce biocompatible hybrid materials [47,48]), for sensing layers and bio-interfaces productions, forecasting feasible applications as biosensors and nanomedicine, including drug delivery and bioimaging. GL layers are produced by a top-down demolition of a nanostructured carbon black (CB) in which the graphenic layers are embedded. The CB is first oxidized with hot nitric acid and the resulting material is then reduced with hydrazine hydrate (two steps oxidation/reduction approach) [45,49]. The synthetic approach adopted for producing the GL layers offers control of the relevant chemical-physical features, and it is suitable for bulk production [45]. The production of GL layers offers several advantages over the conventional methods to produce graphene and graphite oxide from graphite via the typical Hummer–Hoffman and derived approaches since it does not introduce metal contaminants such as manganese ions in the final product, which represent an actual challenge for the scientific community [50] as they are responsible for side toxic effects. Moreover, the selected CB for the GL layers production was purposively chosen among many others, with a negligible level of adsorbed organic materials (as polycyclic aromatic hydrocarbons, PAH) and metal contaminants (<0.1%) limiting the possible presence of potentially toxic heavy metals in the final product (GL layers) [51]. As a matter of fact, the GL layers obtained are metal-free, as confirmed by the XPS survey [46,49].

Differently from GO and reduced GO (rGO), the GL layers are not a single- or few-layer graphene but water-stable short stacked graphenic layers (3–10 stacks on the basis of the Raman survey [45]) with lateral dimension around 50 nm [49]. The small dimensions and the presence of oxygen and nitrogen functional groups (the nitrogen and oxygen percentage contents are 6.1 wt. % and 39.7 wt. %, respectively) limit aggregation phenomena allowing a high colloidal stability in a wide pH range, from 3 to 14 [49] without the use of any surfactant. Zeta potential, indeed, keeps negative (−45 mV) due to the presence of anionic charges on the surface. The oxygen functional groups decorating the graphenic edges are mainly carboxylic and carbonyl groups as depicted by FTIR and X-ray photoemission spectroscopy surveys [45,46]. Coulometric–potentiometric titration in the pH range 2.7–7.0 allowed identifying and quantifying two dominant oxygen functional groups in the carboxylate region with pKa = 3.40 ± 0.05 (number of sites = 900 ± 30 μmol/g) and pKa = 5.5 ± 0.1 (number of sites = 240 ± 30 μmol/g, mainly lactones and carboxylic anhydride groups) [49]. The presence of intact graphenic basal planes within GL layers was suggested by the thermal, optical and electrical conductive properties exhibited by the CB-derived material [45,49].

Thus far, we analyzed the in vitro toxicity of GL layers in mammalian cell cultures, when embedded in eumelanin pigment, reporting their biocompatibility on two different cell typologies; murine embryonic stem cells (ESC) and rat microglial cells (MC) [48]. We also analyzed the bacteriostatic properties of GL layers, demonstrating their ability to act as an inhibitor toward the planktonic growth of *S. aureus* cells, hindering the formation of *S. aureus* biofilms [52]. However, the toxicological profile of GL layers in a vertebrate system has not yet been investigated before. In the present research, we fabricated GL layers from a nanostructured CB and investigated their bio interactions with embryonic zebrafish. Zebrafish are commonly employed as vertebrate models to evaluate the harmful effects of different nanomaterials as their value in toxicology has been proved [53,54]. To the best of our knowledge, this is the first study investigating the in vivo toxicological profile of a GRM produced from a CB. Our reported biocompatibility of GL layers in a vertebrate model will enlarge the potential application of the new class of nanomaterial in the biomedical field.

## 2. Materials and Methods

### 2.1. Materials

#### 2.1.1. Reagents

Analytical grade chemical reagents were purchased from Merck, Darmstadt, Germany, and used as received. Carbon Black (CB) N110 furnace type was obtained from Sid Richardson Carbon Co (Akron, OH, USA). The selected CB is characterized by a H/C atomic ratio of 0.058, a density of 1.8 g/mL at 25 °C and by an inorganic content less than 0.1 wt. %. It is a meso-porous material with a surface area of 139 m^2^/g. Its microstructure is organized in chain-like aggregates with a hydrodynamic diameter, measured by Dynamic Light Scattering (DLS), of 160 ± 20 nm. The diameter of the aggregates building blocks (primary particles or nodules) is 15–20 nm [55].

#### 2.1.2. Gl Layers

GL layers were obtained from CB through a two steps oxidation/reduction approach as previously reported [45,49]. The CB powder was first oxidized with concentrated nitric acid (67 wt. %) at 100 °C under reflux and stirring for 90 h. The resulting hydrophilic precipitate was recovered by centrifugation, washed three times with distilled water and then dried at 105 °C. The oxidized material was then reduced in water by hydrazine hydrate (100 °C, 24 h, reflux), leading to the production of GL layers as black suspension. The GL layers were recovered by filtration on Millipore Durapore^®^ PVDF filter units (pore size 0.22 μm) and carefully washed. Part of the solid recovered by filtration was then resuspended in a proper volume of water to obtain the desired mass concentration (1 g/L) and stored at 4 °C until use.

### 2.2. Material Characterization

#### Atomic Force Microscopy (AFM)

AFM images were acquired on an XE100 Park instrument (Park Systems Corporates, Suwan, Korea) operating in non-contact (NC) mode (amplitude modulation, silicon nitride cantilever from Nanosensor) at room temperature and in ambient conditions. To limit GL layer aggregation, the samples for NC-AFM imaging were prepared by drop-casting a very diluted GL water-suspension (0.1 μg/mL) onto freshly cleaved mica substrates and then allowed to dry in air at room temperature.

### 2.3. Biological Studies

#### 2.3.1. Zebrafish Culture

Adult wild-type (wt) fish were maintained in a circulating system and were fed daily and were fed daily. The water temperature was keep at 28.0 ± 1 °C and the light cycle was set in normal day–night illumination (14 h light:10 h dark).

#### 2.3.2. In Vivo Toxicity

Embryos exhibiting normal development were gathered at 4 h post-fertilization (hpf) and distributed in 24-well plates in standard E3 medium. Embryos were kept at 28 °C and treated with various dilutions of GL layers in E3 medium (5, 10, 50 and 100 μg/mL) and medium without GL layers as the control, until 120 hpf. The biotoxicity of GL layers on zebrafish growth was assessed in terms of swimming distance, heartbeat rate, hatching and survival rates and abnormalities, by observation under a stereomicroscope equipped with a CCD camera [56]. All animal experiments were performed in full compliance with the revised directive 2010/63/EU.

#### 2.3.3. Statistical Analysis

All experiments were carried out in triplicate for statistical analysis. All data were presented as mean ± SD. One-way analysis of variance (ANOVA) in combination with Holm–Šídák post hoc test was used to compare each treatment group with controls. A difference was considered to be statistically significant at *p* < 0.01.

## 3. Results and Discussion

### 3.1. GL Layers Chemico-Physical Characteristics

**Figure 1:**
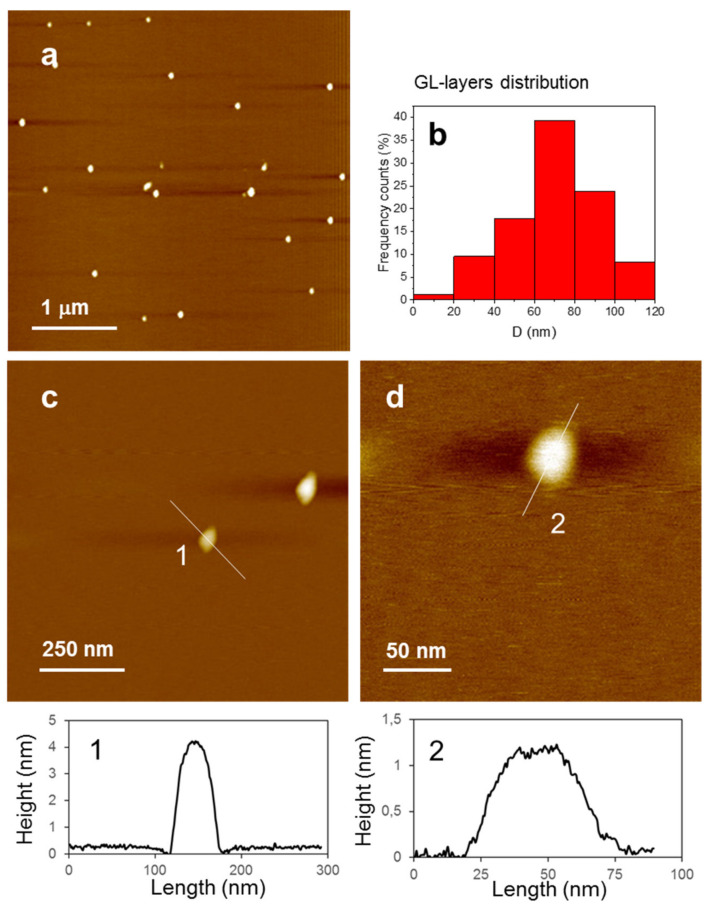
(**a**–**d**) NC-AFM topographic images of GL nanoparticles at different magnifications; (**b**) GL layers dimensional distribution; (**1**–**2**) height profiles taken along the lines highlighted in (**c**,**d**) panels.

The GL layers chemico-physical characteristics were fully assessed in previous studies [45,46,47,48,49] The most relevant GL-layers characteristics are briefly summarized in the Introduction. The batch of GL layers purposively prepared for this study was carefully checked before starting the present study. The AFM images reported in Figure 1a, acquired on an XE100 Park instrument operating in non-contact (NC) mode (amplitude modulation, silicon nitride cantilever from Nanosensor) at room temperature and in ambient conditions, confirms the presence of individual GL layers. Selected height profiles crossing the particles (Figure 1c,d) show vertical sizes ranging from about 1 nm or less to a few nanometers and lateral dimensions of few tens of nanometers (60–70 nm, <50 nm, based on half-height profile reported in Figure 1 for some selected particles).

### 3.2. Biotoxicity Assessment

**Figure 2:**
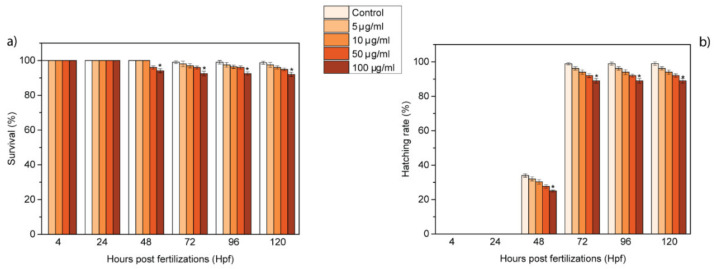
Effects of GL layers on zebrafish development. (**a**) Survival (%) and (**b**) hatching rates (%) of zebrafish treated with GL layers (* *p* < 0.01).

To determine if GL layers had any adverse effects on zebrafish, embryos were exposed to GL layer suspensions (5, 10, 50 and 100 μg/mL) in the E3 medium to allow their uptake. Until 72 hpf, the embryos are surrounded by a protective membrane, named chorion. The GL layers pass the chorion through its pores (0.5–0.6 μm), enter in the embryos and subsequently interact with them. To evaluate the effects of these interactions, several toxicological endpoints were assessed in a temporal window starting from 4 to 120 h post-fertilization (hpf). In particular, the hatching and survival rates were analyzed every 24 h; the measured values are reported in Figure 2. The survival rate presented a low time- and concentration-dependent decrease from 48 to 120 hpf (Figure 2a). The hatching rate also showed a time- and concentration-dependent trend, with a significant difference in comparison with the control for the survival rate from 48 to 120 hpf at 100 μg/mL (Figure 2b). The embryos hatched to larvae in their normal temporal window. The values of survival and hatching rates were not perturbed by treatment with 5–100 μg/mL GL layers, reporting no harmful effects of GL layers in accordance with the OECD guidelines (normative law). The trend of these two end-points completely differs from the one reported for other GRMs [23,24,40]. In fact, other GRMs, such as graphene and graphene oxide, lead to a fast decrease in the survival rate and a delay in the hatching rate, due to their toxicity.

**Figure 3:**
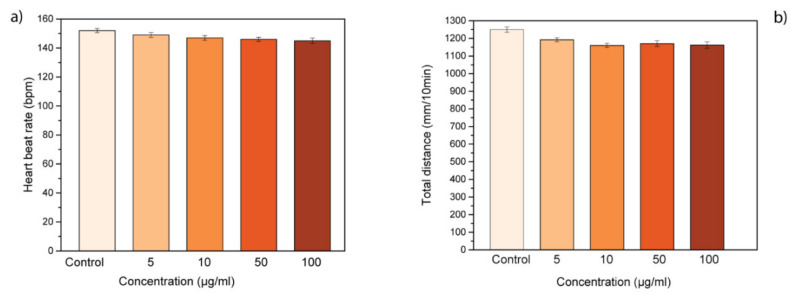
Behavioral effects of GL layers on zebrafish development. (**a**) Heartbeat rate and (**b**) locomotor activity of zebrafish treated with GL layers.

Moreover, the heartbeat rate of treated zebrafish was monitored to evaluate the possible physiological abnormalities induced by GL layers treatment. At the highest concentrations tested, GL layers did not affect the heartbeat rate of exposed larvae. In fact, the heartbeat of treated samples was similar to the control group, treated only with E3 embryo medium (Figure 3a). In addition, we evaluated the locomotor activity of treated larvae at 72 hpf to determine if GL layers could have a continuous influence on larval behavior (Figure 3b). The treatment with different concentrations of GL layers did not lead to hypo- or hyperactivity in the zebrafish larvae in comparison with the control larvae. In summary, GL layers presented no influence on the cardiac and swimming activity of zebrafish larvae with a contrasting trend to other GRMs [23,24,40]. In particular, pristine graphene leads to an alteration of the heartbeat rate and consequently to cardiac defects during embryogenesis.

**Figure 4:**
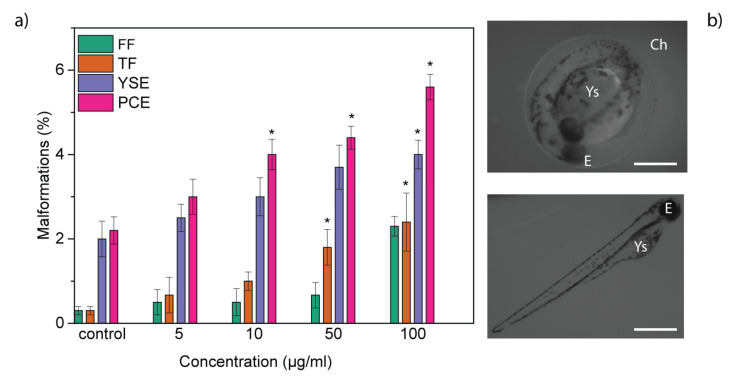
(**a**) Malformations induced in zebrafish exposed to GL layers; (* *p* < 0.01); and (**b**) microscopic images of zebrafish embryo at 48 hpf and larvae at 96 hpf treated with 100 ppm of GL layers. Scale bar = 500 μm. Ch: chorion; E: Eye; Ys: yolk sac.

The organogenesis represents a critical phase of zebrafish growth. During this stage, differentiation of organs occurs, and the high level of proliferation makes them especially incline to abnormalities. For this reason, we evaluated the possible abnormalities induced by GL layers. In treated larvae, typical malformations were observed, including pericardial edema (PCE), yolk sac edema (YSE), tail flexure (TF) and fin fold flexure (FF). However, the incidence of malformations was low (Figure 4a), confirming the biosafety of GL layers. The types of abnormalities observed are the same that are induced by other graphene-related nanomaterials. In fact, different studies have shown that graphene, graphene oxide and nanographene oxide caused morphological defects affecting the eye, the tail, the heart and the yolk sac, with high percentages of malformations [23,24].

Our findings report, for the first time, that GL layers possess good biocompatibility in the embryonic zebrafish vertebrate model, and that they present completely different toxicological profiles in respect to the other members of the GRM family. These results are particularly significant, considering that the GL layers are a new member of the graphene-related nanomaterials family.

Several works have reported the harmful bioeffects in embryonic zebrafish of different members of the graphene-related material (GRM) family [24,25], including graphene [23,27,34], graphene oxide [19,26,28,30,39], nanographene oxide [35] and graphene quantum dots [32]. These graphene-related nanomaterials lead to values of survival and hatching rate rates of ≤90% and ≤80%, respectively, while our GL layers (Figure 2) presented values of ≥90% and ≤80%. Moreover, graphene and graphene oxide caused cardiac defects and behavioral changes in zebrafish while our GL layers did not affect the behavioral and cardiac activities of treated embryos and larvae.

For the first time, we demonstrated the biosafety of the GL layers in a complex vertebrate model. The different endpoints evaluated presented no perturbations in their trends, in contrast with previously studied graphene-related materials, revealing no influence or effects exerted by the GL layers treatments. Given their good biocompatibility and novelty as new members of the GRM family of nanomaterials, GL layers represent a promising platform for biological and biomedical applications.
